# Correction: Kommerein et al. Antiplanktonic and Antibiofilm Activity of *Rheum palmatum* Against *Streptococcus oralis* and *Porphyromonas gingivalis. Microorganisms* 2022, *10,* 965

**DOI:** 10.3390/microorganisms12112141

**Published:** 2024-10-25

**Authors:** Nadine Kommerein, Nina Vierengel, Jonathan Groß, Till Opatz, Bilal Al-Nawas, Lena Katharina Müller-Heupt

**Affiliations:** 1 Department of Prosthetic Dentistry and Biomedical Materials Science, Hannover Medical School, Carl-Neuberg-Straße 1, 30625 Hannover, Germany; kommerein.nadine@mh-hannover.de; 2 Department of Chemistry, Johannes Gutenberg-University, Duesbergweg 10–14, 55128 Mainz, Germany; vierengel@uni-mainz.de (N.V.); jgross03@uni-mainz.de (J.G.); opatz@uni-mainz.de (T.O.); 3 Department of Oral- and Maxillofacial Surgery, University Medical Center Mainz, Augustusplatz 2, 55131 Mainz, Germany; bilal.al-nawas@unimedizin-mainz.de

In the original publication [1], there was a mistake in the legend for Figure 3, subtitle B. The term “increased” should be replaced with “decreased”. The correct legend appears below. The authors state that the scientific conclusions are unaffected. 

The correct legend is as follows:

**Figure 3:** Effects of *R. palmatum* root extract on *S. oralis* and *P. gingivalis* biofilms. After two hours of incubation, viability of *S. oralis* and *P. gingivalis* in 24-h biofilms was significantly inhibited by low concentrations of *R. palmatum* root extract of at least 1.95 mg/L-with a greater decrease in the viability of *P. gingivalis*. (**A**) Treatment with 1.95 mg/L and 31.25 mg/L *R. palmatum* root extract increased biofilm volume of *S. oralis*, whereas biofilm volume was decreased by 500 mg/L *R. palmatum* root extract. (**B**) Treatment with 1.95 mg/L and 500 mg/L *R. palmatum* root extract significantly decreased biofilm volume of *P. gingivalis*. (**C**) Treatment with 1.95 mg/L or more of *R. palmatum* root extract significantly decreased viability of *S. oralis* in biofilms. (**D**) Treatment with 1.95 mg/L or more of *R. palmatum* root extract significantly decreased viability of *P. gingivalis* in biofilms. (**E**) Treatment with 1.95 mg/L and above of *R. palmatum* root extract increased metabolic activity of *S. oralis* in biofilms. (**F**) Treatment with 1.95 mg/L or more of *R. palmatum* root extract increased metabolic activity of *P. gingivalis* in biofilms. N = 6 with *S. oralis* strain ATCC 9811 and *P. gingivalis* strain ATCC 33277/DSM 20709. * *p* < 0.05, ** *p* < 0.01, *** *p* < 0.001, **** *p* < 0.0001.

This correction was approved by the Academic Editor. The original publication has also been updated.
