# Supplementary material for: Antiplanktonic and Antibiofilm Activity of Rheum palmatum Against Streptococcus oralis and Porphyromonas gingivalis
Source: Microorganisms. 2022 May 3;10(5):965. doi: 10.3390/microorganisms10050965 (PMC9143743; doi:10.3390/microorganisms10050965)
Supplement: Supplementary file 1 [file microorganisms-10-00965-s001.zip › microorganisms-1685680-supplementary.pdf]

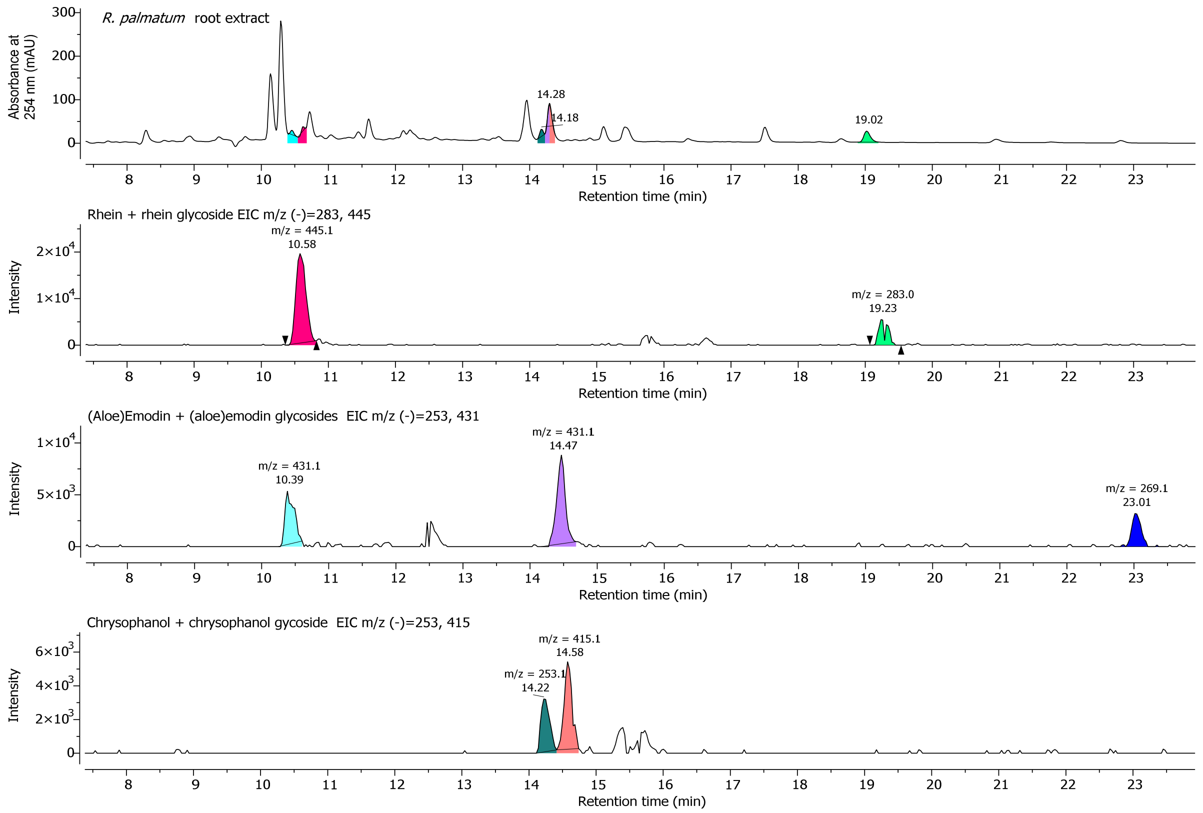

Figure S1 – Extract from HPLC-MS analysis of *R. palmatum*. Shown is the UV chromatogram at 254 nm (top) and the extracted ion current chromatograms (EIC) of the five most abundant anthraquinones and their glycosides in negative ionization mode (ESI –).
